# *In Vitro* Hepatic Metabolism of Curcumin Diethyl Disuccinate by Liver S9 from Different Animal Species

**DOI:** 10.3389/fphar.2020.577998

**Published:** 2020-11-16

**Authors:** Ponsiree Jithavech, Pahweenvaj Ratnatilaka Na Bhuket, Wiwat Supasena, Guanyinsheng Qiu, Shengqing Ye, Jie Wu, Tin Wui Wong, Pornchai Rojsitthisak

**Affiliations:** ^1^ Pharmaceutical Chemistry and Natural Products Program, Faculty of Pharmaceutical Sciences, Chulalongkorn University, Bangkok, Thailand; ^2^ Natural Products for Ageing and Chronic Diseases Research Unit, Chulalongkorn University, Bangkok, Thailand; ^3^ College of Biological, Chemical Sciences and Engineering, Jiaxing University, Jiaxing, China; ^4^ School of Pharmaceutical and Materials Engineering & Institute for Advanced Studies, Taizhou University, Taizhou, China; ^5^ Non-Destructive Biomedical and Pharmaceutical Research Centre, iPROMISE, Universiti Teknologi MARA, Puncak Alam, Malaysia; ^6^ Department of Food and Pharmaceutical Chemistry, Faculty of Pharmaceutical Sciences, Chulalongkorn University, Bangkok, Thailand

**Keywords:** curcumin, curcumin diethyl disuccinate, *In vitro* metabolism, liver S9, monoethylsuccinyl curcumin

## Abstract

Liver S9 (LS9) is a nearly complete collection of all hepatic drug-metabolizing enzymes. It is a low-cost model for predicting drug metabolic activity. This study aimed to identify the suitability of using LS9 of different animal sources in drug metabolism profiling with respect to the possible translation of the *in vitro* outcomes to clinical studies. The *in vitro* hepatic metabolism of curcumin diethyl disuccinate (CDD) in LS9 of rats, dogs, monkeys, and humans was evaluated. The identity of CDD metabolites and the metabolism kinetic parameters, including degradation rate constant, *in vitro*/*in vivo* intrinsic clearance, and half-life, were determined. CDD was rapidly metabolized into monoethylsuccinyl curcumin and curcumin in LS9 of all tested species mainly by carboxylesterases (CESs), including CES1 and CES2, and butyrylcholinesterase. The *in vitro* intrinsic clearance of CDD was in the order of human > dog > monkey > rat, whereas that of monoethylsuccinyl curcumin in the order of dog > monkey > human > rat; this parameter was not correlated with their respective *in vivo* clearance, which followed the order of dog > monkey > rat > human. Therefore, *in vitro* drug metabolism data inferred from LS9 of nonhuman origin, especially from monkeys and dogs, cannot be used as preclinical data for human trials, as humans have a smaller liver-to-body weight ratio than monkeys, dogs, and rats. The *in vivo* drug metabolism is dictated by the anatomical factors of the test subject.

## Introduction

**FIGURE 1:**
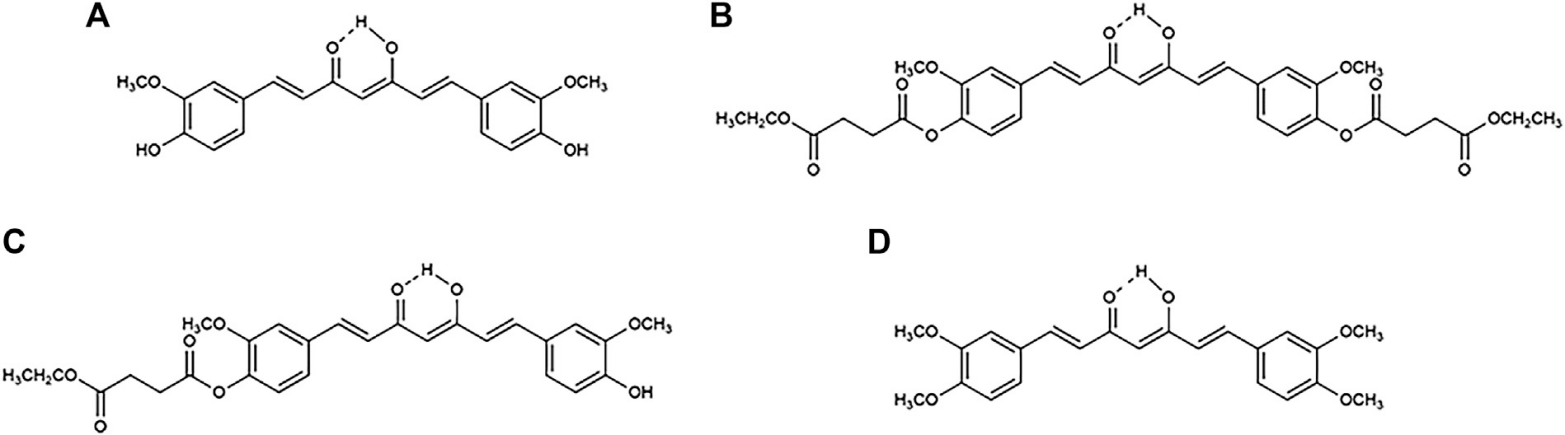
Chemical structures of **(A)** curcumin, **(B)** curcumin diethyl disuccinate (CDD), **(C)** monoethylsuccinyl curcumin (MSCUR), and **(D)** dimethylcurcumin (DMC).

Curcumin (Figure 1A) is a nature-derived polyphenol that possesses various biological activities, such as antioxidant (Calabrese et al., 2008; Khan et al., 2019), anti-inflammatory (Buadonpri et al., 2009; Chaniad et al., 2018; Shimizu et al., 2019), antiviral (Praditya et al., 2019), antitumor (Aggarwal et al., 2003; Muangnoi et al., 2019a; Muangnoi et al., 2019b), and anti-psoriasis (Gomez et al., 2019) properties. Owing to its diverse biological activities and low toxicity, curcumin is an attractive compound for drug development (Strimpakos and Sharma, 2008; Pouliquen, 2014; Ratnatilaka Na Bhuket et al., 2017; Pagano et al., 2018; Rafiee et al., 2019). However, its poor water solubility, chemical and metabolic instability, and low bioavailability pose challenges in its application as a therapeutic agent (Anand et al., 2007; Bangphumi et al., 2016; Toden and Goel, 2017; Dei Cas and Ghidoni, 2019). To overcome these barriers, curcumin has been formulated or covalently designed in the form of nanoparticles (Boonyasirisri et al., 2015; Luckanagul et al., 2018; Gomez et al., 2019), polymer conjugates (Wichitnithad et al., 2011a), and prodrugs (Wichitnithad et al., 2011b; Muangnoi et al., 2018).

Curcumin diethyl disuccinate (CDD; Figure 1B), a succinate ester prodrug of curcumin, has been shown to exhibit a higher level of physicochemical stability in phosphate buffer pH 7.4 than curcumin (Wichitnithad et al., 2011b) without compromising its ability to be converted to its active metabolite in the plasma (Wichitnithad et al., 2011b; Ratnatilaka Na Bhuket et al., 2016; Ratnatilaka Na Bhuket et al., 2019). CDD possesses good *in vitro* and *in vivo* antitumor (Wichitnithad et al., 2011b; Muangnoi et al., 2019a; Muangnoi et al., 2019b), antioxidant (Muangnoi et al., 2019c), and antinociceptive (Wongsrisakul et al., 2010) effects. Further processing of CDD in chitosan–alginate nanoparticles sustains its release for at least 72 h, with enhanced cellular uptake and cytotoxicity in human breast adenocarcinoma (MDA-MB-231) cells (Bhunchu et al., 2015; Bhunchu et al., 2016; Sorasitthiyanukarn et al., 2017). The pharmacokinetics of CDD in rats showed that CDD is rapidly eliminated from the bloodstream following oral and intravenous administration via plasma esterase hydrolysis (Bangphumi et al., 2016). Further, *in vitro* plasma metabolism studies have shown that CDD is rapidly converted to curcumin in the plasma of rats, dogs, and humans, with different hydrolytic rates and various plasma esterases involved (Ratnatilaka Na Bhuket et al., 2019). However, the hepatic metabolism of CDD has yet to be investigated.

Studies on the *in vitro* hepatic drug metabolism provide a controlled experimental platform for predicting the hepatic clearance, bioavailability, potential toxicity, and drug–drug interactions of therapeutics (Wilk-Zasadna et al., 2015; Sloczynska et al., 2019). Recombinant enzymes, liver microsomes, liver S9 (LS9), and hepatocytes are commonly used as *in vitro* drug metabolism models (Dudda and Kürzel, 2013; Costa et al., 2014). LS9 contains both phase I and II metabolic enzymes, including microsomal and cytosolic enzymes, which represent a nearly complete collection of all drug-metabolizing enzymes in the liver (Dudda and Kürzel, 2013; Richardson et al., 2016). It is a low-cost model that has good storage stability (Richardson et al., 2016). LS9 has been found to predict the metabolic activity of drugs with greater accuracy than microsomes (Richardson et al., 2016). The use of human LS9 (HLS9) can reduce prediction bias in high-clearance prodrugs, compared with human hepatocytes (Nishimuta et al., 2014). LS9 contains several esterase enzymes, including carboxylesterases (CESs), acetylcholinesterase (AChE), butyrylcholinesterase (BChE), paraoxonase (PON), and carboxymethylenebutenolidase (CMBL) (Liederer and Borchardt, 2006; Ishizuka et al., 2010; Fukami and Yokoi, 2012; Richardson et al., 2016), of which CESs are the main hydrolytic enzymes of several ester prodrugs, such as sofosbuvir, sacubitril, tenofovir alafenamide, selexipag, telotristat ethyl, and baloxavir marboxil (Najjar and Karaman, 2019). CESs expressed in each mammal species exhibit different enzyme activities and distributions (Hosokawa, 2008; Di, 2019).

One of the advantages of using the LS9 system is that it allows for direct interspecies comparison of the metabolic activity and pathways of ester prodrugs. This aids in the selection of appropriate doses and preclinical species for in vivo experiments, such as pharmacokinetics and toxicity studies (Fu et al., 2016). The use of the LS9 system reduces cost, time, and the number of animals used in drug development. Knowledge of the drug-metabolizing enzymes involved in the biotransformation of a test compound helps in predicting drug–drug interactions. Oseltamivir is metabolized by CES1, but the hydrolysis of oseltamivir can be reduced by clopidogrel, which has also been shown to be the substrate of CES1 (Shi et al., 2006). Evaluation of the hepatic metabolism of CDD using LS9 from several preclinical species and humans is envisaged to facilitate the development of CDD for clinical applications.

In this study, we aimed to identify the suitability of using LS9 of different animal origins in drug metabolism profiling with respect to the possible translation of the *in vitro* outcomes to clinical studies. The *in vitro* hepatic metabolism of CDD in LS9 from four mammal species, including rats, dogs, monkeys, and humans, was investigated with respect to its metabolite formative process and the hepatic esterases involved. We hypothesized that the nature of the metabolites formed, the metabolism rate, and the participating esterases of the CDD metabolism may differ according to the source of LS9. This would imply that the therapeutic efficacy of curcumin, an active metabolite of CDD, may be expressed differently in different animal species and that the enzymatic metabolism of CDD may be dependent on the animal species. Therefore, the adoption of such metabolic outcomes for human clinical evaluation requires careful justification.

## Materials and Methods

### Materials

CDD, monoethylsuccinyl curcumin (MSCUR; Figure 1C), curcumin and dimethylcurcumin (DMC) (internal standard; Figure 1D) were synthesized as previously described (Wichitnithad et al., 2011b; Ratnatilaka Na Bhuket et al., 2019). The purity of all synthesized compounds was >98%, as determined by high-performance liquid chromatography analysis. Acetonitrile (Burdick and Jackson, Korea) and formic acid (Merck, Germany) were the mobile phase used in ultrahigh-performance liquid chromatography (UHPLC), with ultrapure water prepared using the Barnstead MicroPure water purification system (Thermo Scientific, Germany). LS9 originating from human (HLS9; pooled), monkey (MLS9; cynomolgus), dog (DLS9; beagle), and rat (RLS9; Sprague–Dawley) (Invitrogen, United States) was the hepatic enzyme used as the metabolism model. Bis(4-nitrophenyl) phosphate (BNPP), digitonin, phenylmethylsulfonyl fluoride (PMSF), 1,5-bis(4-allyldimethylammoniumphenyl) pentan-3-one dibromide (BW284c51), tetra(monoisopropyl) pyrophosphortetramide (iso-OMPA), 4-(hydroxymercurio) benzoic acid sodium salt (PCMB), 5,5′-dithiobis (2-nitrobenzoic acid) (DTNB) (Sigma-Aldrich, United States), loperamide (Tokyo Chemical Industry, Japan), and ethylenediaminetetraacetic acid (EDTA; Fisher Scientific, Leicestershire, UK) were used as esterase inhibitors. Other chemicals used as supplied were absolute ethanol (Qchemical, Malaysia), potassium phosphate dibasic, and potassium dihydrogen phosphate (Merck).

### Metabolite Identification of Curcumin Diethyl Disuccinate

Metabolite identification of CDD was performed with HLS9, MLS9, DLS9, and RLS9. A stock solution of CDD was prepared at a concentration of 0.5 mM in 50% acetonitrile in water. Each LS9 in phosphate buffer pH 7.4 prepared at a protein concentration of 1 mg/ml was preincubated at 37°C for 5 min. Next, 49 µl of preincubated LS9 was spiked with 1 µl of CDD stock solution to obtain a test sample of 10 µM CDD, which was subsequently incubated at 37°C for 0.5 min. The metabolism reaction of each sample was terminated by adding 100 µl of ice-cold acetonitrile, and the sample was then vortexed and centrifuged at 14,000 rpm (14,488 ×*g*) at 4°C for 10 min. For metabolite identification, 120 µl of the supernatant was diluted with 40 µL of water before analysis. The metabolites and CDD in the supernatant were identified against CDD, MSCUR, and curcumin standards using liquid chromatography–quantitative time-of-flight tandem mass spectrometry (LC-QTOF-MS/MS) in a Thermo Dionex UltiMate 3000 RSLCnano system equipped with an LPG Micro Pump (Thermo Scientific, United States) and MicrOTOF-Q II (Bruker, Germany). Using this system, the accurate masses for both parent and fragment ions were evaluated to obtain the elemental formula of each compound.

The LC-QTOF-MS/MS conditions were set as previously reported (Ratnatilaka Na Bhuket et al., 2019). Briefly, the chromatographic separation was performed on a C18 column (2.1 × 50 mm, 1.7 µm; Acquity UPLC^®^ BEH, Waters, United States), which was maintained at 35°C. The isocratic mobile phase was composed of solvents (A) acetonitrile and (B) water with 0.2% (v/v) formic acid delivered at a flow rate of 120 μl/min with an injection volume of 15 µl. The positive electrospray ionization (ESI+) source parameters were set as follows: nebulization nitrogen gas pressure, 2.5 bar; capillary voltage, −4.5 kV; dry nitrogen flow rate, 8 L/min; dry nitrogen temperature, 220°C; and end plate offset voltage, −500 V. MS/MS spectra were acquired at a collision energy of 20 eV and over a range of m/z 50–1,000. System control, data acquisition, and data processing were performed using Bruker Compass Data Analysis 4.0 software, and Bruker SmartFormula software was used to determine the molecular formula of the analytes.

### *In Vitro* Metabolism Profiling of Curcumin Diethyl Disuccinate

The metabolism profiles of CDD were investigated against HLS9, MLS9, DLS9, and RLS9. A stock solution of CDD was prepared at 150 µM in 50% acetonitrile in water before use. The metabolism study was initiated by spiking 735 µl of preincubated LS9 with 15 µl of CDD stock solution, and the mixture was subsequently incubated at 37°C. The final concentrations of CDD and LS9 were 3 μM and 1 mg/ml, respectively. A 50-µl aliquot from the mixture was collected at 0.5, 1, 2, 3, 4, 5, 10, 15, 30, 45, 60, 90, and 120 min and immediately mixed with 100 µl of ice-cold acetonitrile containing 0.45 µM of internal standard DMC to terminate the metabolic reaction. The mixture was vortex-mixed and centrifuged at 14,000 rpm (14,488 ×*g*) at 4°C for 10 min. For the analysis of the metabolism profile, 120 µl of the supernatant was diluted with 40 µl of water prior to UHPLC (Agilent series 1290; Agilent Technology, United States) analysis. The sample at the initial time point (0 min) was prepared as the other samples, except that heat-inactivated HLS9, MLS9, DLS9, and RLS9 (80°C for 20 min; 1 mg/ml protein) were used.

The UHPLC system consisted of an Agilent series 1290 quaternary pump with an online degasser, autosampler, column oven, and diode array detector. The mobile phase was composed of (A) 0.1% (v/v) formic acid in acetonitrile and (B) 0.1% (v/v) formic acid in water. Each solution was filtered through a nylon filter (pore size, 0.22 µm; MS^®^ Membrane Filters; Membrane Solutions, United States) before use. The mobile phase flow rate was kept at 1.2 ml/min in a gradient mode using the following elution program: 0–3.0 min, initial A-B (40:60, v/v); 4.5–7.0 min, isocratic elution A-B [50:50, (v/v); 8.5–11.0 min, isocratic elution A-B (60:40, v/v); and 12.0–17.0 min, isocratic elution A-B (40:60, v/v)]. CDD, curcumin, and MSCUR in the supernatant were partitioned using a C8 column (4.6 mm × 50 mm, 2.7 µm; HALO, Advanced Materials Technology, United States) with reference to DMC. The detection wavelength was set at 400 nm (*λ*_max(CDD)_ = 400 nm, *λ*_max(curcumin)_ = 427 nm, *λ*_max(MSCUR)_ = 418 nm, and *λ*_max(DMC)_ = 421 nm). The column was maintained at 35°C throughout the run, and the supernatant injection volume was set at 20 µl.

The concentrations of CDD, curcumin, and MSCUR in the supernatant were determined from the standard plots, which were prepared by spiking individual working solutions of the compounds to heat-inactivated HLS9, MLS9, DLS9, and RLS9 (Alcorn et al., 2007; Montoro-Garcia et al., 2009; Singh et al., 2010). These working standards were extracted with 100 µl of ice-cold acetonitrile containing 0.45 µM of DMC and then 120 μl of the supernatant was diluted with 40 μl of water prior to the UHPLC analysis. The peak area ratios of CDD, curcumin, and MSCUR to the internal standard were computed against linear concentration ranges of 0.025–4.5 µM CDD, 0.025–4.5 µM curcumin, and 0.0125–0.75 µM MSCUR, respectively, with the coefficient of determination (r^2^) values greater than 0.99.

The percent content of CDD and the metabolites formed upon its incubation with LS9, namely, curcumin and MSCUR, was plotted against incubation time. Analysis of metabolite profiles suggested that the metabolism of CDD adopted a two-step, consecutive, first-order irreversible reaction, a basic model for prodrug metabolism (Cho and Yoon, 2018), as shown in Eq. 1:

$$A\;\overset{k_{1}}{\to }\;B\;\overset{k_{2}}{\to }\;C,$$ (1)

where A = CDD, B = MSCUR, and C = curcumin.

The following equations were adopted to describe the changes in CDD and MSCUR content over time (t):

$$A\;=\;A_{0}e^{-k_{1}t}$$ (2)

$$B=\;\frac{k_{1}A_{0}}{k_{2}-k_{1}}\left(e^{-k_{1}t}-e^{-k_{2}t}\right),$$ (3)

where A_0_ is the CDD concentration at time 0, *k*_*1*_ is the degradation rate constant of CDD, and *k*_*2*_ is the degradation rate constant of MSCUR. *k*_1_ and *k*_2_ were determined by using **Eqs. 2 and 3**, respectively, with nonlinear regression analysis using the SOLVER function of Microsoft Excel for Office 365 MSO (16.0.12527.20986) (Brown, 2001). The half-life (*t*_1/2_) was calculated using Eq. 4:

$$t_{1/2}=\frac{0.693}{k}\;$$ (4)

The *in vitro* intrinsic clearance (CL_int,*in vitro*_) of CDD and MSCUR in LS9 was calculated using Eq. 5:

$$CL_{int,\;in\;vitro}=\;k\;\times \;\frac{{\left[V\right]}_{incubation}\left(\text{ml}\right)}{{\left[P\right]}_{incubation}\left(\text{mg protein}\right)},$$ (5)

where [*V*]_incubation_ is the incubation volume in ml, and [*P*]_incubation_ is the amount of LS9.

The *in vivo* intrinsic clearance (CL_int,*in vivo*_) was modeled from CL_int,*in vitro*_ values using physiologically based scaling factors, as described by Eq. 6:

$$CL_{int,\;in\;vivo}=\;CL_{int,\;in\;vitro}\;\times \;S9\;\left(\text{mg}\;\text{protein}/\text{g}\;\text{liver}\right)\;\times \;\frac{Liver\;weight\;\left(\text{g}\right)}{Body\;weight\;\left(\text{g}\right)},$$ (6)

where S9 is the amount of S9 protein per gram of liver of preclinical species that was assumed to be the same as that reported for humans (121 mg/g) (Nishimuta et al., 2014). The liver weight per body weight of humans, monkeys, dogs, and rats was 25.7, 30.0, 32.0, and 40.0 g/kg, respectively (Davies and Morris, 1993). The total *in vivo* intrinsic clearance (CL_int,total*in vivo*_) was the sum of CL_int,*in vivo*(CDD)_ and CL_int,*in vivo*(MSCUR)._

The predicted hepatic clearance was calculated using the well-stirred model (Hosea et al., 2009), as described by Eq. 7:

$$CL_{H}=\frac{Q_{H}\times \;CL_{int,\;in\;vivo\;total}\;\times fu_{p}\;}{Q_{H}\;+\;\left(CL_{int,\;in\;vivo\;total}\;\times \;fu_{p}\right)},$$ (7)

where Q_H_ = hepatic blood flow of 20, 44, 40, and 70 ml min^−1^ kg^−1^ in humans, monkeys, dogs, and rats, respectively (Hosea et al., 2009; Zhang et al., 2018). fu_p_ (fraction of unbound CDD in plasma), estimated at 0.0594, was derived from Eq. 8 (Gleeson, 2007; Muangnoi et al., 2019b):

$$\log\;K\;=\;\;\log\;\left(\frac{1\;-\;fu_{p}}{fu_{p}}\right),$$ (8)

where log K is the transformation of fu_p_ into a pseudo-equilibrium constant. CDD is a neutral compound with a log P of 2.55 (Muangnoi et al., 2019b). The log K value for a neutral compound with log P < 3 is approximately 1.2 (Gleeson, 2007). The fu_p_ was 0.0594. More than 90% of CDD was bound to plasma proteins, indicating that CDD had a high protein-binding property.

### Identification of the Hepatic Esterases Responsible for Curcumin Diethyl Disuccinate Hydrolysis

The hepatic esterases involved in the hydrolytic cleavage of CDD in the respective LS9 samples were identified by the chemical inhibition approach with nine esterase inhibitors, namely, BNPP, digitonin, loperamide, PMSF, BW284c51, iso-OMPA, PCMB, DTNB, and EDTA. Each esterase inhibitor (5 µl) at a final concentration of 100 μM, except for EDTA at a concentration of 1 mM, was incubated individually with 44 µl of each LS9 (HLS9, MLS9, DLS9, and RLS9; 0.02 mg/ml in phosphate buffer pH 7.4) for 30 min at 37°C prior to the addition of 1 µl CDD (150 µM) to obtain a final concentration of 3 µM. The mixture was then incubated at 0.5 min for MLS9 and DLS9 or at 1 min for HLS9 and RLS9. The metabolism reaction was terminated by adding 100 µl of ice-cold acetonitrile. The mixture was then vortexed and centrifuged at 14,000 rpm (14,488 ×*g*) at 4°C for 10 min. For hepatic esterase identification, 120 µl of the supernatant was diluted with 40 µl of water prior to the UHPLC analysis of CDD and curcumin. The inhibitor-free control (incubation of CDD in the absence of inhibitors), enzyme-free control (incubation of CDD in the absence of LS9), and organic solvent-free control (incubation of CDD in the absence of organic solvent in the inhibitor solution) were similarly evaluated.

The curcumin formation rate and relative curcumin formation rate were calculated using **Eqs. 9 and 10**, as follows (Bisswanger, 2014; Chanteux et al., 2014; Thomsen et al., 2015):

$$Curcumin\;formation\;rate\;\left(\text{nmol}\;{\text{min}}^{-1}\;{\text{mg}}^{-1}\right)\;=\;\;\frac{Curcumin\;content\;\left(\text{µM}\right)\;-\;Enzyme-free\;control}{Incubation\;time\;\times \;LS9\;protein\;content\;in\;mixture}$$ (9)

$$Relative\;curcumin\;formation\;rate\;\left(\%\right)=\frac{Curcumin\;formation\;rate\;}{Curcumin\;formation\;rate\;of\;inhibitor-free\;control}\times 100\;$$ (10)

Enzymatic inhibition was classified as strong, moderate, weak, or no inhibition based on a relative curcumin formation rate of <20%, 20–49%, 50–80%, and >80%, respectively (Food Drug Administration Center for Drug Evaluation Research, 2012).

### Statistical Analysis

At least three replicates were conducted for each experiment. Statistical analyses were performed using one-way analysis of variance followed by Dunnett’s test using IBM SPSS Statistics for Windows, version 22.0 (IBM Corp., Armonk, NY, United States) (Chanteux et al., 2014).

## Results and Discussion

### Identification of Curcumin Diethyl Disuccinate Metabolites

**FIGURE 2:**
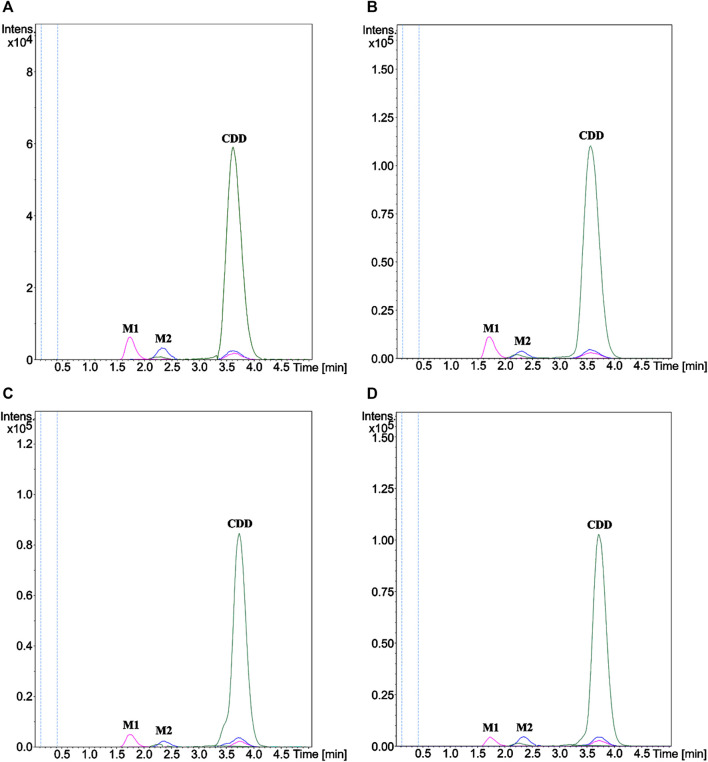
Extracted ion chromatograms of CDD and its metabolites M1 and M2 in **(A)** HLS9, **(B)** MLS9, **(C)** DLS9, and **(D)** RLS9. CDD (10 µM) was incubated in HLS9, MLS9, DLS9, and RLS9 (1 mg/ml) at 37°C for 0.5 min.

The structures of the metabolites formed through incubation of CDD in HLS9, MLS9, DLS9, and RLS9 were elucidated using LC-QTOF-MS/MS and confirmed against synthetic standard compounds. The extracted ion chromatograms (Figure 2) showed peaks at 3.6 min (m/z 625.2280) that corresponded to the CDD standard (m/z 625.2282) as well as two unknown peaks, designated as metabolite 1 (M1) and metabolite 2 (M2), which had retention times of approximately 1.7 and 2.3 min, respectively. Mass spectrometry of M1 resulted in molecular ions [M1 + H^+^] of m/z 369.1327, 369.1331, 369.1336, and 369.1332 with reference to HLS9, MLS9, DLS9, and RLS9 (Table 1). However, M2 had molecular ions [M2 + H^+^] of m/z 497.1809, 497.1811, 497.1797, and 497.1809 with reference to HLS9, MLS9, DLS9, and RLS9, respectively. The results indicated that CDD can be metabolized into M1 and M2 in S9 from all four tested species.

**TABLE 1:** LC-QTOF-MS/MS data of CDD and its hydrolyzed metabolites detected after incubation in human, monkey, dog, and rat LS9.

Id	Species	Retention time (min)	Elemental composition	Theoretical mass (m/z)	Measured mass (m/z)	Mass error (ppm)	Product ions (m/z)
CDD	Standard	3.60	C_33_H_37_O_12_	625.2280	625.2282	0.4	129.0533, 177.0543, 369.1292, 497.1820
Curcumin	Standard	1.70	C_21_H_21_O_6_	369.1333	369.1331	0.6	177.0547, 285.1136
MSCUR	Standard	2.32	C_27_H_29_O_9_	497.1806	497.1807	0.2	129.0532, 177.0543, 369.1338
M1	HLS9	1.74	C_21_H_21_O_6_	369.1333	369.1327	1.7	177.0550, 285.1140
	MLS9	1.71	C_21_H_21_O_6_	369.1333	369.1331	0.4	177.0563, 285.1145
	DLS9	1.74	C_21_H_21_O_6_	369.1333	369.1336	1.0	177.0574, 285.1147
	RLS9	1.73	C_21_H_21_O_6_	369.1333	369.1332	0.2	177.0569, 285.1122
M2	HLS9	2.32	C_27_H_29_O_9_	497.1806	497.1809	0.5	129.0542, 177.0538, 369.1324
	MLS9	2.30	C_27_H_29_O_9_	497.1806	497.1811	1.0	129.0543, 177.0572, 369.1344
	DLS9	2.35	C_27_H_29_O_9_	497.1806	497.1797	1.8	129.0537, 177.0539, 369.1313
	RLS9	2.34	C_27_H_29_O_9_	497.1806	497.1809	0.5	129.0546, 177.0540, 369.1364

The MS/MS spectrum data of M1 and M2 showed different fragmentation patterns, as summarized in Table 1. The fragmentation of M1 yielded similar product ions at m/z 177 and 285, regardless of the source of S9. M2 had a fragmentation pattern similar to that of product ions at m/z 129, 177, and 369 for all four sources of S9. To confirm the identity of M1 and M2, curcumin and MSCUR were synthesized and used as standard compounds. The retention times of M1 and M2 were similar to those of curcumin and MSCUR standards, respectively (Table 1). In addition, the MS/MS spectra of M1 and M2 generated the same molecular ions and fragmentation patterns as those of curcumin and MSCUR, respectively. M1 and M2 were therefore confirmed as curcumin and MSCUR, respectively. The difference in the m/z value between the CDD parent compound (m/z 625) and the curcumin metabolite (m/z 369) corresponded to the loss of m/z 256 attributed to hydrolysis of the diethylsuccinyl group adjacent to both phenolic functional groups. For the MSCUR metabolite (m/z 497), the loss of m/z 128 from the parent compound corresponded to the loss of mono-ethylsuccinyl moiety from CDD. The metabolite identification results indicated the formation of curcumin and MSCUR as metabolites following the CDD metabolism in LS9 from all tested species.

### *In Vitro* Metabolism Profiling of Curcumin Diethyl Disuccinate

In this study, the *in vitro* metabolism profiles of CDD were determined using LS9 from different animals to determine the species-dependent characteristics. The LS9 of dog, monkey, and rat was selected in addition to HLS9, on the note that such animals exhibit comparable metabolism profiles to humans, where the major metabolites potentially present in humans can be found in these animals to a similar extent (Luffer-Atlas and Atrakchi, 2017).

In general, the drug concentration range used in the metabolism study was 1–3 μM, as it is expected to be much lower than Km (Michaelis constant, the substrate concentration that yields a half-maximal velocity) (Riley and Grime, 2004; Baranczewski et al., 2006). In the present study, 1 µM of CDD was not used, as this resulted in insufficient sensitivity for the detection of the intermediate metabolite (MSCUR) by UHPLC-UV. On the contrary, MSCUR was traceable when 3 µM of CDD was used. Previous studies on ester-based drug metabolisms using the LS9 system have adopted substrate concentrations between 2 and 10 µM (Khojasteh et al., 2008; Murakami et al., 2014). CDD had very short half-lives in LS9 from all tested species (Table 2). It was rapidly hydrolyzed in LS9. The use of 3 µM CDD was unlikely to saturate the esterases in LS9 and thus deemed appropriate.

**TABLE 2:** Kinetic parameters of hepatic hydrolysis of CDD and MSCUR in human, monkey, dog, and rat LS9.

LS9 origin				
Parameter	Human	Monkey	Dog	Rat
*k*_1__(CDD)_ (min^−1^)	1.51 ± 0.22	0.81 ± 0.13	0.85 ± 0.29	0.68 ± 0.13
*k*_2__(MSCUR)_ (min^−1^)	12.07 ± 0.86	20.79 ± 3.13	24.61 ± 2.55	9.66 ± 0.60
*t*_1/2 (CDD)_ (min)	0.46 ± 0.06	0.87 ± 0.13	0.87 ± 0.25	1.04 ± 0.18
*t*_1/2 (MSCUR)_ (min)	5.76 ± 0.42 × 10^–2^	3.39 ± 0.54 × 10^–2^	2.84 ± 0.30 × 10^–2^	7.19 ± 0.44 × 10^–2^
CL_int,_ _*in vitro*(CDD)_ (ml min^−1^ mg protein^−1^)	1.51 ± 0.22	0.81 ± 0.13	0.85 ± 0.29	0.68 ± 0.13
CL_int,_ _*in vitro*(MSCUR)_ (ml min^−1^ mg protein^−1^)	12.07 ± 0.86	20.79 ± 3.13	24.61 ± 2.55	9.66 ± 0.60
CL_int,_ _*in vivo*(CDD)_ (L h^−1^ kg^−1^)	282.18 ± 41.74	176.96 ± 27.49	198.61 ± 66.73	197.51 ± 37.52
CL_int,_ _*in vivo*(MSCUR)_ (L h^−1^ kg^−1^)	2,251.20 ± 161.06	4,527.30 ± 682.15	5,716.98 ± 592.58	2,805.37 ± 174.76
CL_int,_ _*in vivo*_ _total_ (L h^−1^ kg^−1^)	2,533.38 ± 124.30	4,704.26 ± 695.64	5,915.59 ± 535.14	3,002.88 ± 180.63
CL_H_ (L h^−1^ kg^−1^)	1.19 ± 4.61 × 10^–4^	2.61 ± 3.87 × 10^–3^	2.38 ± 1.48 × 10^–3^	4.10 ± 5.77 × 10^–3^

**FIGURE 3:**
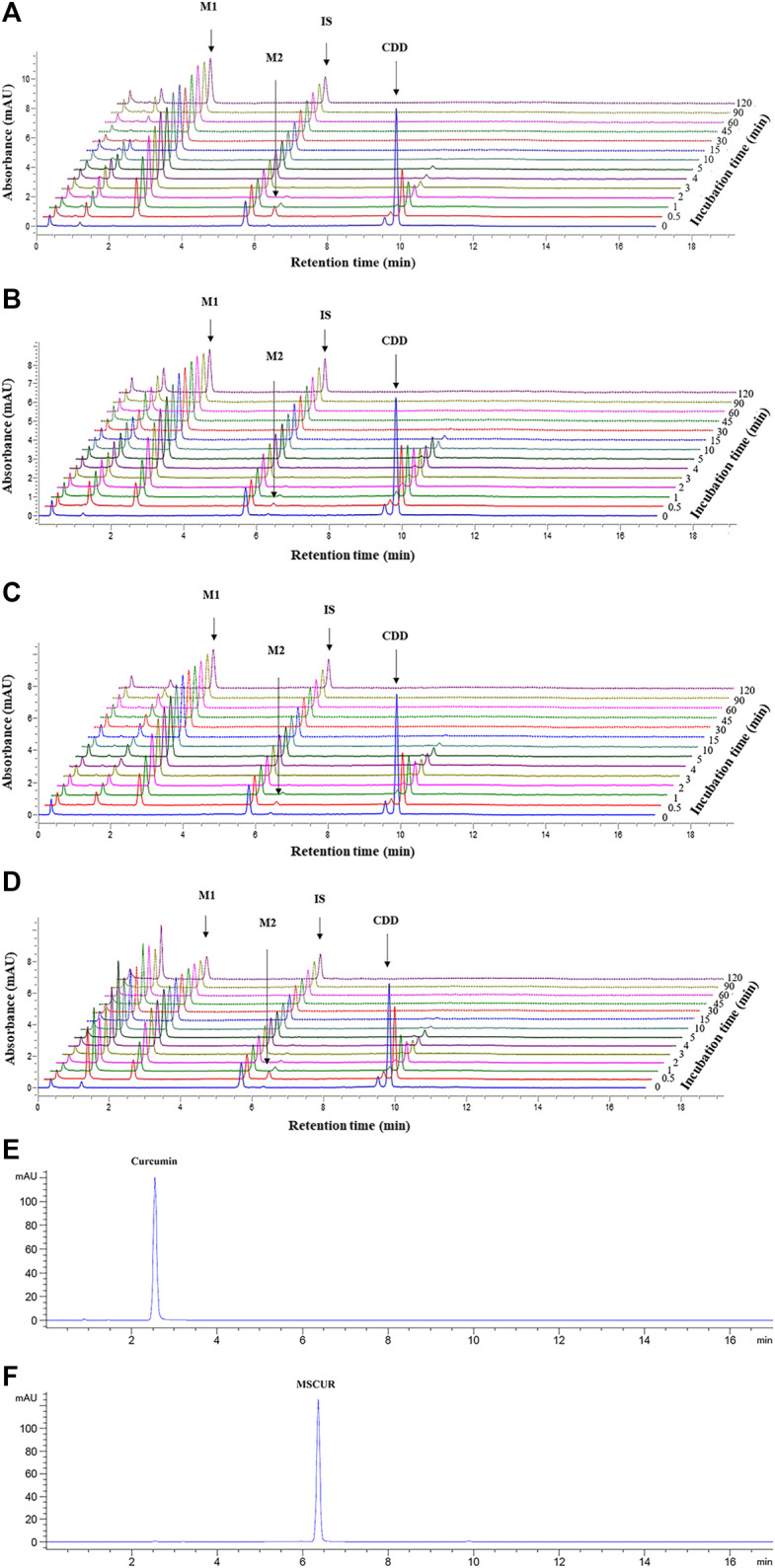
UHPLC chromatograms of CDD and its metabolites in **(A)** HLS9, **(B)** MLS9, **(C)** DLS9, and **(D)** RLS9 as a function of incubation time. CDD (3 µM) was incubated with HLS9, MLS9, DLS9, and RLS9 (1 mg/ml) at 37°C. **(E,F)** are UHPLC chromatograms of standard curcumin and MSCUR, respectively.

The metabolite profiles of CDD in LS9 from all tested species as a function of incubation time are shown in UHPLC chromatograms (Figures 3A–D). The retention times of CDD and DMC internal standards were 9.8. and 5.7 min, respectively. The peak with a retention time of 9.5 min (Figure 3) represented a tautomer of CDD, as it exhibited the same mass-to-charge ratio as CDD (m/z 625.2279, mass error = 0.1 ppm), which was inferred from studies by Kawano et al. (2013), Ratnatilaka Na Bhuket et al. (2016), Jia et al. (2017), and Ratnatilaka Na Bhuket et al. (2019). Two metabolites with retention times of 2.5 and 6.3 min were formed and referred to as M1 and M2, respectively. Subsequent studies revealed that M1 and M2 corresponded to curcumin and MSCUR standards, respectively, which were eluted at similar retention times as the metabolites (Figures 3E,F). UHPLC results confirmed the identity of M1 and M2, in agreement with the LC-QTOF-MS/MS results, as described in “*Identification of Curcumin Diethyl Disuccinate Metabolites*” (Figure 2).

**FIGURE 4:**
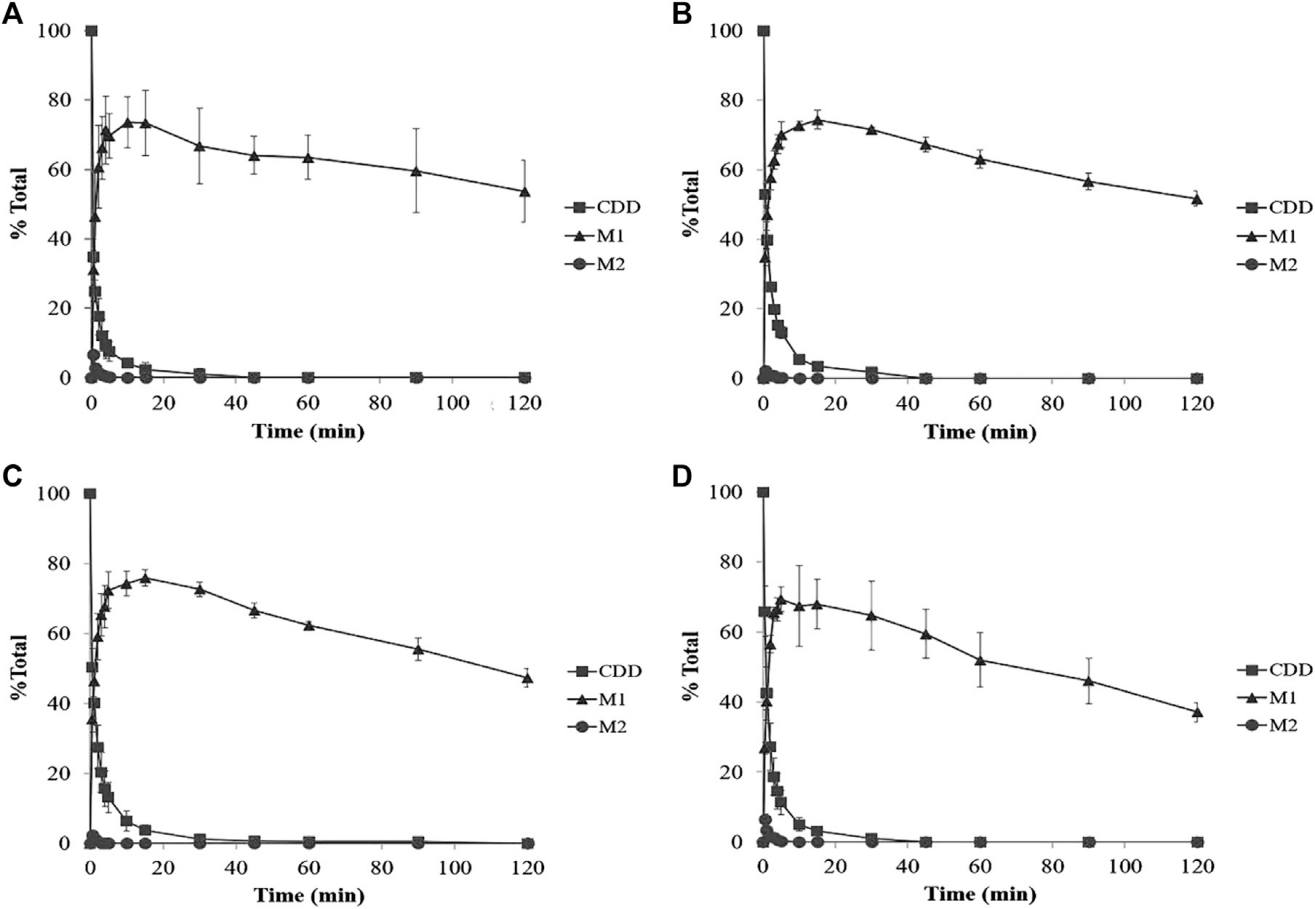
Percent CDD and its metabolite time profiles in **(A)** HLS9, **(B)** MLS9, **(C)** DLS9, and **(D)** RLS9 as a function of incubation time. The mixtures containing CDD (3 µM) and HLS9, MLS9, DLS9, and RLS9 (1 mg/ml) were incubated at 37°C.

The metabolism profiles of CDD were similar in LS9 from the four tested species (Figure 4). The depletion of CDD in LS9 from all tested species showed that M1 was the major metabolite and that M2 was formed in a small quantity. After the addition of CDD in LS9, CDD was rapidly and completely degraded, in which more than 95% of the CDD was hydrolyzed within the first 15 min of incubation. The major metabolite, M1 or curcumin, was produced in nearly 70% of each LS9 following 15 min of incubation. The amount of curcumin declined by 20% and 30% over 15–120 min, as previously reported by Wang et al. (1997) and Wichitnithad et al. (2011b). The maximum amount of M2 or MSCUR was approximately 10% at 0.5 min of incubation and declined thereafter. The results suggested that CDD was rapidly metabolized in LS9 from all tested species, with curcumin as the major product and MSCUR as the intermediate metabolite.

The metabolism kinetic parameters including the *k*_1_, *k*_2_, *t*_1/2_ of CDD [*t*_1/2(CDD)_]; *t*_1/2_ of MSCUR [*t*_1/2(MSCUR)_]; CL_int,_ _*in vitro*_ of CDD [CL_int,_ _*in vitro*(CDD)_]; CL_int,_ _*in vitro*_ of MSCUR [CL_int,_ _*in vitro*(MSCUR)_]; CL_int,_ _*in vivo*_ of CDD [CL_int,_ _*in vivo*_ _(CDD)_]; CL_int,_ _*in vivo*_ of MSCUR [CL_int,_ _*in vivo*_ _(MSCUR)_]; and CL_int,_ _*in vivo total*_ were determined, and the results are summarized in Table 2. In all LS9, *k*_2_ values were 10- to 20-fold higher than *k*_1_ values, indicating that *k*_1_ was the rate-determining parameter. Both CDD and MSCUR were rapidly metabolized by all LS9s, with *t*_1/2_ of less than 1.04 and 7.19 × 10^–^^2^ min, respectively. The short half-lives of CDD and MSCUR observed in this study were consistent with those of other ester-containing compounds that underwent enzymatic hydrolysis, such as vicagrel (*t*_1/2_ of approximately 1.6 min) (Qiu et al., 2016) and GS-6620 (*t*_1/2_ of approximately 4.0 min) (Murakami et al., 2014).

Interspecies differences were observed in CL_int,_ _*in vitro*(CDD)_ and CL_int,_ _*in vitro*(MSCUR)_ (Table 2). The CL_int,_ _*in vitro*(CDD)_ values obtained from different LS9 were in the following order: human > dog > monkey > rat. The CL_int,_ _*in vitro*(CDD)_ in HLS9 was higher than that in DLS9, MLS9, and RLS9 by 1.8-, 1.9-, and 2.2-fold, respectively. In the case of MSCUR, its CL_int,_ _*in vitro*_ _(MSCUR)_ values followed the order of dog > monkey > human > rat. Unlike that of CDD, the CL_int,_ _*in vitro*(MSCUR)_ values of DLS9 and MLS9 were approximately two- and 1.7-fold higher than that of HLS9. The CL_int,_ _*in vitro*(MSCUR)_ value of RLS9 was 0.8-fold lower than that of HLS9. Bioinformatic analysis suggested that the amino acid sequences and structural homology of metabolic enzymes are dependent on the animal species (Satoh and Hosokawa, 1998; Taketani et al., 2007; Hosokawa, 2008). This may confer the differences in the activities and substrate specificity of enzymes of different species origins (Taketani et al., 2007; Williams et al., 2011; Ma et al., 2017; Wang et al., 2020).

CL_int,_ _*in vivo*(CDD)_ and CL_int,_ _*in vivo*(MSCUR)_ were predicted using a physiologically based scaling factor (Table 2). The predicted *in vivo* hepatic elimination of CDD, expressed by the sum of the *in vivo* clearance of CDD and MSCUR metabolite (CL_int,_ _*in vivo*_ _total_), was in the following order: DLS9 > MLS9 > RLS9 > HLS9. Apparently, the *in vitro* hepatic clearances of CDD and MSCUR in DLS9, MLS9, and RLS9 were parallel to the predicted *in vivo* total clearance profiles. HLS9, on the other hand, was correlated with a relatively small *in vivo* total clearance, despite its relatively high *in vitro* CDD clearance, and had higher *in vitro* MSCUR clearance than RLS9. This could be due to the fact that the ratio of liver mass to body mass of humans is lower than that of rats. Using the well-stirred model, the order of CL_H_ values in HLS9, MLS9, DLS9, and RLS9 did not correspond to the CL_int,_ _*in vivo*_ _total_ in any specific manner. Larger hepatic blood flow was translated to higher CL_H_ level. Hepatic blood flow might have governed the hepatic metabolism of CDD. The interplay between hepatic blood flow and enzymes requires further study.

### Esterases Involved in Curcumin Diethyl Disuccinate Hydrolysis in Liver S9

Earlier, we have optimized the incubation time and LS9 concentrations to investigate the effects of esterase inhibitors on the hydrolytic activity of LS9 enzymes. CDD (3 μM) was used to ensure that the reaction was independent of the substrate concentration, and the formation of curcumin over the incubation time was dependent on the level of active enzymes. The kinetics of CDD depletion in different LS9s are summarized in Supplementary Table S1. The linear time-dependent formation of curcumin in 0.02 mg/ml LS9 was observed at 0–2 min of incubation in LS9 from all tested species (*R* ^2^ > 0.98) (Supplementary Figure S1). The appropriate time point for the enzyme inhibition assay should have a CDD depletion of less than 30% (Food Drug Administration Center for Drug Evaluation Research, 2012; Bisswanger, 2014), and the % CDD depletion should be similar among species to reduce bias due to unequal remaining substrate content (Deng et al., 2018). In addition, the incubation time for the assay should be within the time period that yields linear time-dependent curcumin formation (Bisswanger, 2014). To meet the above criteria, incubation times of 0.5 min for MLS9 and DLS9 (with 25% and 23% CDD depletion, respectively) and 1.0 min for HLS9 and RLS9 (with 19% and 22% CDD depletion, respectively) were selected for the enzyme inhibition assay.

**FIGURE 5:**
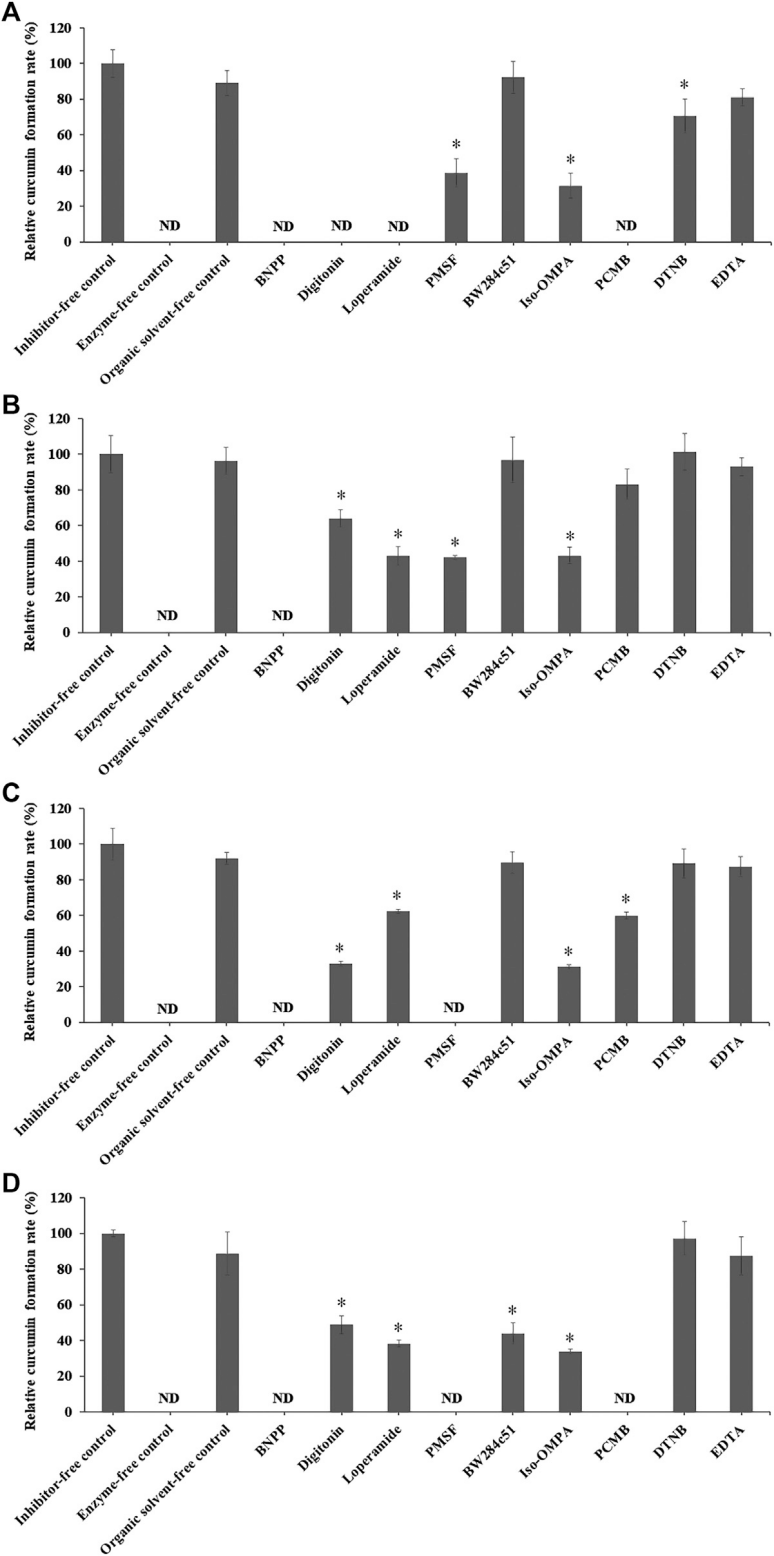
Relative curcumin formation rate of CDD (3 µM) incubated with **(A)** HLS9, **(B)** MLS9, **(C)** DLS9, and **(D)** RLS9 (0.02 mg/ml) in the presence of different inhibitors. Control was prepared by incubating CDD in LS9 without inhibitors. One-way ANOVA analysis with Dunnett’s *post hoc* test: ND = not determined because the curcumin concentration was below the lower limit of quantification; *statistically significant vs. control *p* < 0.001.

Esterase inhibitors, including BNPP, digitonin, loperamide, PMSF, BW284c51, iso-OMPA, PCMB, DTNB, and EDTA are known to inhibit CESs (Chanteux et al., 2014), CES1 (Shimizu et al., 2014), CES2 (Chanteux et al., 2014), serine hydrolase (Abe et al., 2015), acetylcholinesterase (AChE) (Chanteux et al., 2014), butyrylcholinesterase (BChE) (Chanteux et al., 2014), carboxymethylenebutenolidase (CMBL) (Ishizuka et al., 2010), cysteine hydrolase (Bull et al., 1982; Chanteux et al., 2014), and paraoxonase (PON) (Liederer and Borchardt, 2006), respectively. The different effects of these esterase inhibitors on CDD hydrolysis in LS9s of various species origins were investigated (Ma et al., 2017), and the results are summarized in Figure 5.

In the absence of inhibitors, the rates of curcumin formation in HLS9, MLS9, DLS9, and RLS9 were 10.77 ± 0.82, 14.60 ± 1.51, 26.79 ± 2.43, and 12.71 ± 0.25 nmol min^−1^ mg^−1^ protein, respectively, with a relative curcumin formation rate of 100%. The organic solvent did not exhibit any effect on the degradation profiles of CDD into curcumin. A relative curcumin formation rate of 100% was attained in the organic solvent–free control. These results indicated that CDD was degraded via enzymatic hydrolysis in LS9 of all test species.

In humans, CDD hydrolysis in HLS9 was inhibited by BNPP, digitonin, loperamide, and PCMB, and to a lesser extent by PMSF, iso-OMPA, and DTNB. The esterases of HLS9 responsible for CDD hydrolysis were primarily CESs (including CES1 and CES2) and CMBL, and partially BChE. AChE and PON did not play a critical role in the hydrolysis of CDD by HLS9. CESs and CMBL play an essential role in the hydrolysis of prodrugs in the liver, as CESs are primal enzymes expressed in the hepatic microsome, whereas CMBL is highly expressed in the hepatic cytosol of human and preclinical species (Ishizuka et al., 2013; Wang et al., 2018; Di, 2019). The involvement of CESs and CMBL in the ester hydrolysis of CDD in HLS9 is consistent with the ester hydrolysis of other ester prodrugs, such as oseltamivir, methylphenidate, and olmesartan medoxomil (Ishizuka et al., 2013; Laizure et al., 2013).

CDD hydrolysis in MLS9 was inhibited by BNPP, and to a lesser extent by digitonin, loperamide, PMSF, and iso-OMPA. BW284c51, PCMB, DTNB, and EDTA showed no enzyme inhibitory effects. The esterases of MLS9 responsible for the hydrolysis of CDD were CESs (including CES1 and CES2) and BChE. In the case of DLS9, the relative contribution of esterases in the hydrolysis of CDD was as follows: CES1 and CES2 > BChE > CMBL. With reference to RLS9, the relative contribution of esterases in the hydrolysis of CDD was in the following order: CES1 and CES2, ≈CMBL > BChE > AChE.

CDD can be hydrolyzed by CESs (including CES1 and CES2), AChE, BChE, and CMBL. These enzymes are expressed in the liver of human and preclinical species, and they belong to the α/β hydrolase fold family, which may have broad and overlapping substrate specificities (Holmquist, 2000; Liederer and Borchardt, 2006; Ishizuka et al., 2013). CESs, AChE, BChE, and CMBL are known to be involved in the bioactivation of various ester prodrugs. For example, irinotecan (CPT-11), an anticancer prodrug of SN-38, can be transformed into its active metabolite by both CESs and BChE (Liederer and Borchardt, 2006). For the bioactivation of CDD, an ester prodrug of curcumin, the common ester hydrolytic enzymes in LS9 of all tested species are CESs (including CES1 and CES2) and BChE, with CESs having a major contribution in the hepatic hydrolysis of CDD.

Apart from the liver, the gut is another site that plays an important role in the hydrolysis of orally administered ester-based drugs (Nishimuta et al., 2014; Di, 2019). CESs are the major enzymes responsible for CDD hydrolysis. However, the difference in the expression profiles of CES1 and CES2 isoforms between the liver and gut results in different extents of CDD hydrolysis. Our results showed that CDD was metabolized primarily by CES1, which is a predominant isoform in the liver (Laizure et al., 2013; Wang et al., 2018; Di, 2019). However, the involvement of CES1 and CES2 in the gut metabolism of CDD may be different from that of the hepatic metabolism, as CES2 is the major isoform in the small intestine (Laizure et al., 2013; Wang et al., 2018; Di, 2019). In addition to hydrolysis, other metabolic pathways may also play a role in the overall metabolism of CDD and its metabolites. It has been previously shown that tetrahydrocurcumin, curcumin glucuronide, and tetrahydrocurcumin glucuronide are significant metabolites of curcumin (Pan et al., 1999; Ireson et al., 2002; Hoehle et al., 2006; Pfeiffer et al., 2007). MSCUR and curcumin generated from CDD hydrolysis may undergo reduction and glucuronidation. The alternative metabolic pathways and gut metabolism warrant further investigation.

In summary, this study provided comparative data on the hepatic metabolism of CDD in LS9 from rats, dogs, monkeys, and humans. The experiments showed that CDD was rapidly metabolized into MSCUR and curcumin in LS9 from all tested species mainly by CESs, including CES1 and CES2, and BChE. Nevertheless, the hepatic clearances were different between species, which was probably caused by the differences in the expression level and amino acid sequences of esterases as a function of the species origin. Additional studies including gut metabolism, phase I and II metabolisms of CDD metabolites, and the extent of protein binding should be further investigated to map the metabolism profiles of CDD.

## Conclusion

In this study, we investigated the *in vitro* hepatic metabolism of LS9, a nearly complete collection of all hepatic drug-metabolizing enzymes from different animal species. CDD was rapidly metabolized into MSCUR and curcumin in HLS9, MLS9, DLS9, and RLS9 mainly by CESs (CES1 and CES2) and butyrylcholinesterase. The *in vitro* intrinsic clearance of CDD was in the following order: human > dog > monkey > rat LS9. The *in vitro* intrinsic clearance of MSCUR followed the order of dog > monkey > human > rat LS9. The *in vitro* intrinsic clearance was not correlated with the *in vivo* clearance of CDD and MSCUR*,* with the order of dog > monkey > rat > human LS9. *In vitro* drug metabolism data inferred from LS9 of nonhuman origin, especially of monkey and dog origins, cannot be used as preclinical data for human trials. Humans are characterized by smaller liver-to-body weight ratio than that of monkeys, dogs, and rats. Smaller liver-to-body weight ratio translates to lower *in vivo* CDD and MSCUR intrinsic clearance. An *in vitro* LS9 metabolism study does not consider the anatomical factors of the test subject and is not reflective of the *in vivo* metabolic outcome.

## Data Availability Statement

No additional dataset is available for this study. The raw data supporting the conclusions of this article will be made available by the authors, without undue reservation.

## Author Contributions

PJ designed the research, carried out the *in vitro* metabolism study, analyzed the data, and wrote the manuscript. PRB synthesized the compounds and wrote the manuscript. WS conducted the LC-QTOF-MS/MS analysis. GQ, SY, JW, and TWW reviewed and edited the manuscript. PR designed the research and reviewed and edited the manuscript.

## Funding

This research was supported by a scholarship from the Graduate School of Chulalongkorn University, the 100th Anniversary Chulalongkorn University Fund for Doctoral Scholarship, the 90th Anniversary Chulalongkorn University Fund (Ratchadaphiseksomphot Endowment Fund, No. GCUGR1125613043D), and the Chulalongkorn Academic Advancement into its Second Century (CUAASC) Project.

## Conflict of Interest

The authors declare that the research was conducted in the absence of any commercial or financial relationships that could be construed as a potential conflict of interest.
