# Supplementary material for: In Vitro Hepatic Metabolism of Curcumin Diethyl Disuccinate by Liver S9 from Different Animal Species
Source: Front Pharmacol. 2020 Nov 16;11:577998. doi: 10.3389/fphar.2020.577998 (PMC7703437; doi:10.3389/fphar.2020.577998)

**Supplementary section**

*In Vitro* Hepatic Metabolism of Curcumin Diethyl Disuccinate by Liver S9 from Different Animal Species

Ponsiree Jithavech^1,2^, Pahweenvaj Ratnatilaka Na Bhuket^2^, Wiwat Supasena^2^, Guanyinsheng Qiu^3^, Shengqing Ye^4^, Jie Wu^4^, Tin Wui Wong^5^, Pornchai Rojsitthisak^2,6*^

^1^ Pharmaceutical Chemistry and Natural Products Program, Faculty of Pharmaceutical Sciences, Chulalongkorn University, Bangkok, Thailand

^2^ Natural Products for Ageing and Chronic Diseases Research Unit, Chulalongkorn University, Bangkok, Thailand

^3^ College of Biological, Chemical Sciences and Engineering, Jiaxing University, Jiaxing, Zhejiang, China

^4^ School of Pharmaceutical and Materials Engineering & Institute for Advanced Studies, Taizhou University, Taizhou, China

^5^ Non-Destructive Biomedical and Pharmaceutical Research Centre, iPROMISE, Universiti Teknologi MARA, Malaysia

^6^ Department of Food and Pharmaceutical Chemistry, Faculty of Pharmaceutical Sciences, Chulalongkorn University, Bangkok, Thailand

**^*^ Corresponding author**

Pornchai Rojsitthisak, Ph.D.

Department of Food and Pharmaceutical Chemistry,

Faculty of Pharmaceutical Sciences, Chulalongkorn University,

254 Phayathai Road, Patumwan, Bangkok 10330 Thailand

Tel.: +66-2-218-8310; Fax: +66-2-254-5195; E-mail: pornchai.r@chula.ac.th

**Identification of optimized incubation condition for enzyme inhibition assay**

The metabolism profiles of CDD and curcumin formation profile were investigated against HLS9, MLS9, DLS9 and RLS9. A stock solution of CDD was prepared at 150 µM in 50% acetonitrile in water before use. The metabolism study was initiated by spiking a 588 µl pre-incubated LS9 with 12 µl CDD stock solution, and subsequently incubated at 37°C. The final concentrations of CDD and LS9 were 3 µM and 0.02 mg/ml, respectively. A 50 µl aliquot from the mixture was collected at 0.5, 1, 2, 3, 4, 5, 10, 15, 30, 45 and 60 min and was immediately mixed with 100 µl of ice-cold acetonitrile containing 0.45 µM of internal standard DMC to terminate the metabolic reaction. The mixture was vortex-mixed and centrifuged at 14,000 rpm (14,488 g) at 4°C for 10 min. Samples of 120 µl of supernatant wee diluted with 40 µl of water prior to UHPLC analysis (Agilent series 1290, Agilent Technology, USA) by means of protocol outlined in Materials and Methods:*“In vitro Metabolism Profiling of Curcumin Diethyl Disuccinate”*. The sample at 0 min was prepared as other samples except that heat-inactivated HLS9, MLS9, DLS9 and RLS9 (80°C for 20 min; 1 mg/ml protein) were used. Duplicates were conducted for each experiment.

The peak area ratios of CDD and curcumin to internal standard in the supernatant were determined. The percent contents of CDD and curcumin were plotted against incubation time. The depletion of CDD was calculated with reference to the initial concentration. The saturated substrate was defined as % depletion less than 30.

The appropriate time point for the enzyme inhibition assay should have the CDD depletion of less than 30 % (Food Drug Administration Center for Drug Evaluation Research, 2012; Bisswanger, 2014), and the % CDD depletion should be similar among species to reduce the bias from unequal remaining substrate content (Deng et al., 2018). In addition, the incubation time for the enzyme inhibition assay should be within the time period that yields the linear time-dependent curcumin formation (Bisswanger, 2014).

**Results**

The kinetics of the CDD depletion in different LS9 were summarized in Table S1. The linear time-dependent formation of curcumin in LS9 of 0.02 mg/ml were observed at 0-2 min of incubation in LS9 of all test species (R^2^ > 0.98) (Figure S1). To meet the above criteria, we therefore selected the incubation time of 0.5 min for MLS9 and DLS9 (with 25 % and 23 % CDD depletion, respectively) and at 1.0 min for HLS9 and RLS9 (with 19 % and 22 % CDD depletion, respectively) for the enzyme inhibition assay. The enzyme concentration of all test species was 0.02 mg/ml.

**Reference:**

1. Bisswanger, H. (2014). Enzyme assays. Perspectives in Science 1, 41-55. doi:10.1016/j.pisc.2014.02.005
2. Deng, Y., Butré, C.I., and Wierenga, P.A. (2018). Influence of substrate concentration on the extent of protein enzymatic hydrolysis. International Dairy Journal 86, 39-48. doi:10.1016/j.idairyj.2018.06.018
3. Food Drug Administration Center for Drug Evaluation Research (2012). *Guidance for industry: drug interaction studies—study design, data analysis, implications for dosing, and labeling recommendations (draft guidance).* Silver Spring, MD; US FDA.

Table S1. The depletion of CDD in HLS9, MLS9, DLS9 and RLS9 against time. CDD at 3 μM were incubated in duplicate of each LS9 (0.02 mg/ml).

| **Species** | **Time**  **(min)** | **% CDD remaining** | **%Depletion of CDD** |
| --- | --- | --- | --- |
| Human | 0  0.5  1^a^  2  3  4  5 | 100.00  82.88  80.95  68.18  57.46  43.15  36.37 | 0  17.12  19.05  31.82  42.54  56.85  63.63 |
| Monkey | 0  0.5^a^  1  2  3  4  5 | 100.00  75.32  63.90  49.34  37.51  28.36  21.92 | 0  24.68  36.10  50.66  62.49  71.64  78.08 |
| Dog | 0  0.5^a^  1  2  3  4  5 | 100.00  76.77  66.99  49.79  42.26  31.30  25.06 | 0  23.23  33.01  50.21  57.74  68.70  74.94 |
| Rat | 0  0.5  1^a^  2  3  4  5 | 100.00  82.81  78.14  62.71  56.54  46.44  40.58 | 0  17.19  21.86  37.29  43.46  53.56  59.42 |

^a^ denotes the selected incubation time with less than 30 % CDD depletion in the LS9 of each test species.

Figure S1. Formation of curcumin in (A) HLS9, (B) MLS9, (C) DLS9 and (D) RLS9, plotted against time. CDD at 3 µM was incubated in duplicate with LS9 of 0.02 mg/ml.


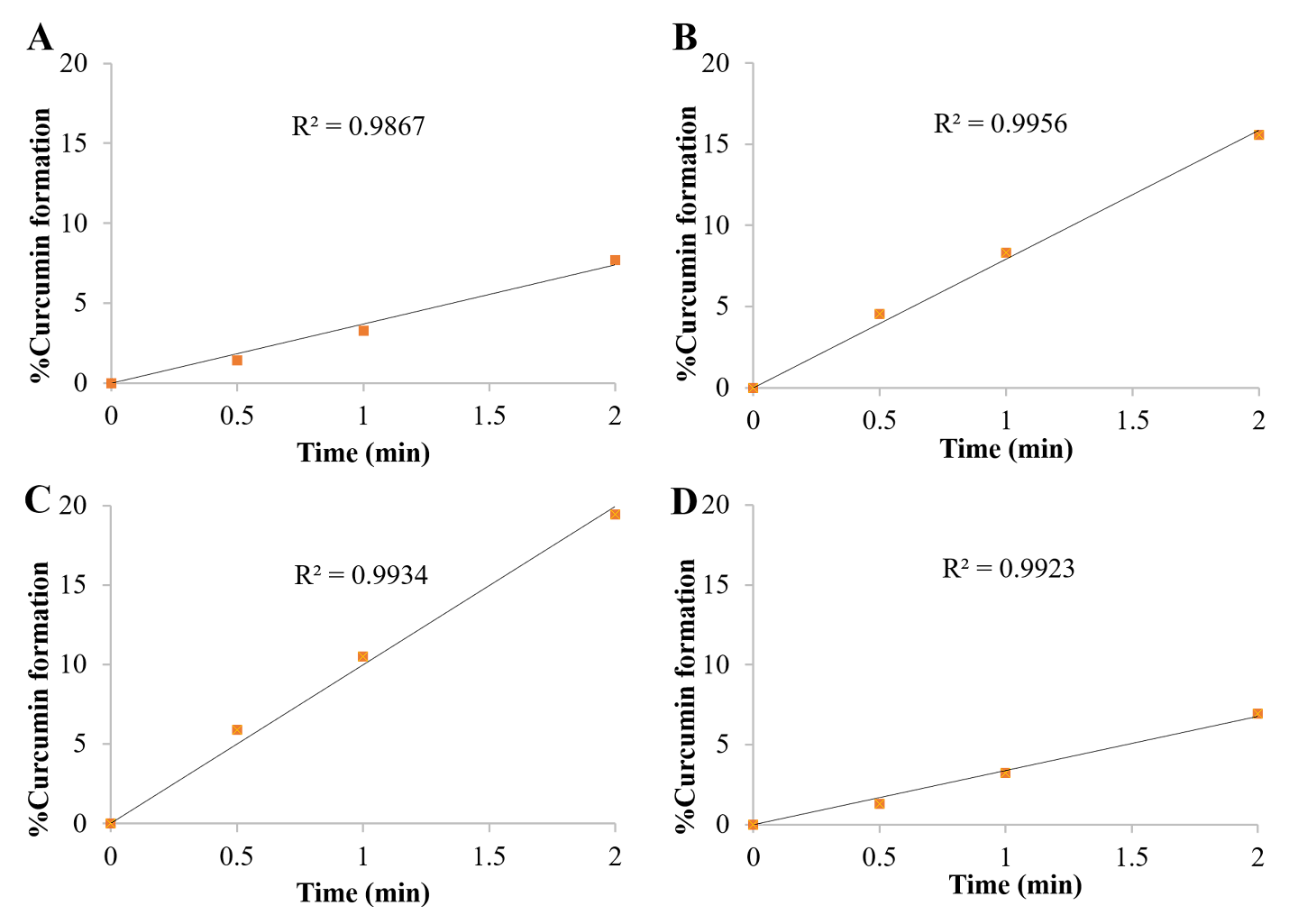

Supplement: Supplementary file 1 [file datasheet1.docx]
